# Physical health symptoms reported by trafficked women receiving post-trafficking support in Moldova: prevalence, severity and associated factors

**DOI:** 10.1186/1472-6874-12-20

**Published:** 2012-07-26

**Authors:** Siân Oram, Nicolae V Ostrovschi, Viorel I Gorceag, Mihai A Hotineanu, Lilia Gorceag, Carolina Trigub, Melanie Abas

**Affiliations:** 1 Health Services and Population Research, Institute of Psychiatry at Kings College London, London, UK; 2 N. Testemitanu Medical and Pharmaceutical University, Chisinau, Republic of Moldova; 3 International Organization for Migration, Chisinau, Republic of Moldova; 4 PROGenEVA, Chisinau, Republic of Moldova

## Abstract

**Background:**

Many trafficked people suffer high levels of physical, sexual and psychological abuse. Yet, there has been limited research on the physical health problems associated with human trafficking or how the health needs of women in post-trafficking support settings vary according to socio-demographic or trafficking characteristics.

**Methods:**

We analysed the prevalence and severity of 15 health symptoms reported by 120 trafficked women who had returned to Moldova between December 2007 and December 2008 and were registered with the International Organisation for Migration Assistance and Protection Programme. Women had returned to Moldova an average of 5.9 months prior to interview (range 2-12 months).

**Results:**

Headaches (61.7%), stomach pain (60.9%), memory problems (44.2%), back pain (42.5%), loss of appetite (35%), and tooth pain (35%) were amongst the most commonly reported symptoms amongst both women trafficked for sexual exploitation and women trafficked for labour exploitation. The prevalence of headache and memory problems was strongly associated with duration of exploitation.

**Conclusions:**

Trafficked women who register for post-trafficking support services after returning to their country of origin are likely to have long-term physical and dental health needs and should be provided with access to comprehensive medical services. Health problems among women who register for post-trafficking support services after returning to their country of origin are not limited to women trafficked for sexual exploitation but are also experienced by victims of labour exploitation.

## Background

Human trafficking is a human rights violation and a serious form of crime which involves the recruitment and movement of individuals – most often by force, coercion or deception – for the purpose of exploitation [1-3]. Trafficking for sexual exploitation is the most commonly recognized form of this crime, but men, women and children are also trafficked for exploitation in a range of labour settings, including agriculture, factory work and domestic servitude, as well as for begging and for forced marriage [4]. Between 2000 and 2010, the International Organisation for Migration (IOM) provided assistance to trafficked persons on 46,554 occasions, including 5,911 instances of assistance in 2010. 43% of people assisted by IOM had been trafficked for sexual exploitation, 33% for labour exploitation (including domestic servitude and begging), 4% for both sexual and labour exploitation, and 20% for other or unknown purposes [5].

Studies from around the world report that trafficked people are often subject to extreme forms of physical, sexual and psychological abuse and to neglect and deprivation [6-10]. Women’s experiences during their exploitation may have multiple physical health consequences. Although physical health is a broad concept [11], potential outcomes include physical injury and pain; neurological, gastrointestinal, gynaecological, dermatological, cardiovascular, and musculoskeletal complications; cognitive and sensory problems; exhaustion and malnutrition; infection; and the deterioration of pre-existing conditions [12]. Yet, there has been limited research on the physical health problems associated with human trafficking [13]. We are aware of only two studies which report on the physical health symptoms experienced by women who have been trafficked for sexual exploitation and of no studies that report on the physical health problems associated with trafficking for labour exploitation [8,14].

Research is also lacking on how trafficked people’s physical health needs vary in relation to demographic variables or characteristics of their trafficking experiences. Studies have shown, however, that trafficked women’s mental health may vary in relation to type of exploitation, duration of exploitation, and time since trafficking [15,16] and that the risk of HIV infection may be associated with age, area of origin, destination, and duration of exploitation [17,18]. Such evidence will be important in informing the development of policy and service approaches for supporting the health and recovery of trafficked people.

This paper 1) describes the physical health symptoms reported by trafficked women receiving assistance from the IOM Assistance and Protection Programme in Moldova; and 2) analyses variation in the prevalence of trafficked women’s reported physical health symptoms by age group, country of destination, type of exploitation, duration of exploitation, and time since returning to Moldova.

## Methods

Survey interviews were conducted with a consecutive sample of trafficked Moldovan women who had registered with the IOM Assistance and Protection Programme (APP) between December 2007 and December 2008 and had participated in a crisis intervention assessment within 5 days of registration. APP support generally comprises crisis intervention care (including a medical, psychological, legal and social needs assessment, and residential care of up to 1 month, which can be extended) followed by a community-based reintegration program (often including social assistance and vocational training) that lasts, on average, 12 months.

### Sample

**Figure 1:**
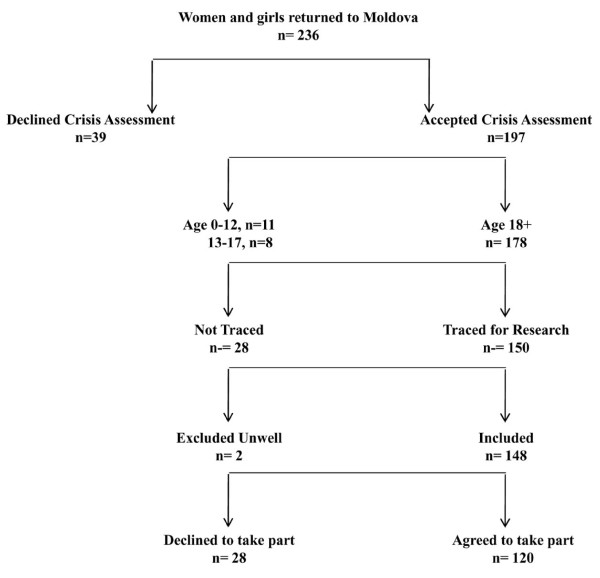
**Recruitment of women into the study from all the women and girls who returned to Moldova through IOM Assistance and Protection services from December 2007 to December 2008.** Originally published in Ostrovschi et al, 2011 (reproduced with permission).

Women were eligible for inclusion if they (i) were aged 18 or over; (ii) were originally resident in Moldova; (iii) had returned to Moldova in the past 2-12 months following a trafficking experience outside of Moldova; and (iv) had registered with IOM in Moldova as a survivor of trafficking and participated in a crisis-intervention assessment within 5 days of registering [19]. Women who had returned to Moldova after working overseas in the sex industry but who had not been trafficked were not eligible for inclusion in the study. Whether or not a woman had been trafficked was determined by IOM case managers. The IOM defines trafficking in accordance with the UN Optional Protocol to Prevent, Suppress and Punish Trafficking in Persons, especially Women and Children [3]. Between 2000 and 2008 the IOM have supported 2,340 trafficked women who returned to Moldova. Women access IOM support after being repatriated by overseas IOM missions or by partner non-governmental organisations (NGOs); following contact with anti-trafficking telephone hotlines; or after being referred by police departments, social protection services or NGOs. A small number of women self-refer for IOM support. Approximately 80% of returning women accept the acute crisis intervention and/or the rehabilitation program. 178 adult women registered with the IOM and participated in crisis assessment during the study period (Figure 1). Eligible women were approached by an IOM social worker and informed of the study aims and the subject matter. Social workers were able to trace 150 of these women, 2 of whom were excluded because of physical illness. Of the 148 women invited to take part in the study, 28 (18.9%) refused to participate.

We followed the World Health Organization Ethical and Safety Recommendations for Interviewing Trafficked Women [20] and complied with the IOM Data Protection Principles [21]. Ethical approval for the study was granted by the Kings College Research Ethics Committee (CREC/07/08-56) and from the N. Testemitanu State Medical and Pharmaceutical University Institutional Review Board. The voluntary nature of participation was emphasised and all women provided written informed consent to participate in the study. Women were excluded if the social worker or research psychiatrist considered them to be too distressed or unwell to take part in the study.

### Measures

Data on socio-demographic variables were available from IOM records and included information on marital status before trafficking, employment status prior to trafficking, and age on return from trafficking. IOM provided restricted access to anonymised and aggregated data on women who did not take part in the study to enable broad comparisons between participants and non-participants. Very little difference was observed between the socio-demographic characteristics of participants and non-participants. No data were available, however, to support comparisons between the women who did and did not register with the IOM assistance programme.

The presence and severity of health symptoms were measured using a shortened (15 item) version of the Miller Abuse Physical Symptoms and Injury Survey (MAPSAIS) [22], a self-report scale that was designed for measuring the long-term health consequences of violence and which has previously been adapted for use with trafficked women [8]. The questions used are presented in Additional file 1. Briefly, women were asked whether they had experienced 15 physical health symptoms in the previous two weeks and, if so, whether they had been “not at all”, “a little”, “quite a bit”, or “extremely” bothered or caused pain by each symptom. The symptoms included on the survey instrument represented a range of physical health domains (e.g. cardiovascular, gastrointestinal, urogenital) and had previously been shown to be prevalent among trafficked women awaiting deportation and receiving crisis-stage post-trafficking support [8,14]. It was not feasible to conduct a comprehensive health assessment and we did not collect data on the presence of chronic conditions (e.g. asthma, diabetes, hypertension) and did not ask women about their pre-trafficking physical or mental health status.

For ethical reasons, we did not collect data on the nature of women’s trafficking experiences, including with regards to how they had been trafficked and how they had left the trafficking situation. If women chose to disclose this information the researchers were trained to listen to them sensitively and non-judgmentally, emphasize that they were not to blame for their experiences, and encourage them to speak with their support worker. Data on country trafficked to, duration of trafficking, type of exploitation, and time since return to Moldova were collected by IOM during registration in Moldova.

### Analysis

Descriptive statistics were calculated for demographic characteristics and variables relating to women’s experiences of trafficking. The prevalence and severity of 15 self-rated physical symptoms were calculated for the total sample. Symptoms were considered to be present if women reported that they had been bothered or caused pain by them a little, quite a bit, or extremely over the past two weeks. Variation relating to demographic and trafficking characteristics (specifically, age group, destination country, type of exploitation, and the duration of exploitation) was assessed by bivariate logistic regression for the 4 most commonly reported symptoms. These demographic and trafficking variables were chosen because previous research with trafficked people had identified them as risk factors for poor health [15,17,18]. The variables “duration of exploitation” and “time since returned to Moldova” were examined as categorical variables in descriptive analyses to show their distribution and the prevalence of physical symptoms within discrete periods. In regression analyses, however, they were treated as continuous variables. All analyses were carried out in STATA version 10 [23].

## Results

The sample comprised 120 women, who ranged in age from 18 to 44 (mean 25.4, SD 5.97) at the time of interview. Table 1 presents the socio-demographic characteristics of the sample; a fuller description is presented elsewhere [18]. Despite most women having completed at least compulsory lower secondary education (88.2%), 68% had been unemployed prior to being trafficked. Over half (54.2%) reported having experienced either sexual abuse or severe physical abuse as children. The majority of women had been trafficked for sexual exploitation (80.8%). Of those who had been trafficked for labour exploitation, 16 had been trafficked for domestic work, 4 for agricultural labour, and 3 for begging. 67.5% of women were exploited for longer than six months, with 27.5% trafficked for longer than a year (mean 9.6 months, SD 5.6 months, range 2-31 months). 39.7% women were trafficked for exploitation in Turkey, 27.5% in Russia, 11.6% in the EU and 21.2% elsewhere (including Bosnia and Herzegovina, Croatia, Israel, Kosovo, Serbia, Ukraine, and the UAE). 61.7% of women had returned to Moldova less than six months prior to interview (mean 5.9 months, SD 3.2 months, range 2-12 months). Information on time since exiting trafficking was not, however, available for analysis. At the point of registration with IOM Moldova, 11.7% of women were married or cohabiting and over half (51.7%) had living children.

**Table 1:** Background characteristics of the sample (n = 120)

**Characteristic**	**% (n)**
***Pre-trafficking***	
*Education status*	
Primary education or less	11.6 (14)
Lower secondary education (9 years)	62.5 (75)
Upper secondary or higher	25.7 (31)
*Employment status*	
Unemployed	68.3 (82)
Unqualified work	20.8 (25)
Student/vocational training	5.8 (7)
Qualified work	5.0 (6)
*Living situation*	
Alone	41.2 (50)
With parents	17.5 (21)
With child(ren)	20.8 (25)
With partner	10.8 (13)
Other	9.2 (11)
*Sexual or severe physical child abuse*	
Yes	54.2 (65)
No	45.8 (55)
***Trafficking***	
*Country trafficked to*	
Russia	28.3 (34)
Turkey	38.3 (46)
Other§	33.3 (40)
*Type of exploitation*	
Sexual	80.8 (97)
Labour	19.2 (23)
*Duration of trafficking (months)*	
1-3	9.2 (11)
4-6	23.3 (28)
7-12	40.0 (48)
13-24	25.0 (30)
>24	2.5 (3)
***Post-trafficking***	
*Age*	
18-20	17.5 (21)
21-25	49.2 (59)
26-30	11.7 (14)
31-44	21.7 (26)
*Employment status*	
Unemployed	36.7 (44)
Unqualified work	30.8 (37)
Student/vocational training	20.8 (25)
Qualified work	11.7 (14)
*Marital status*	
Single	68.3 (82)
Married/cohabiting	11.7 (14)
Separated/divorced/widowed	20.0 (24)
*Has children*	
Yes	51.7 (62)
No	48.3 (58)
*Time since return to Moldova (months)*	
1-3	29.2 (35)
4-6	32.5 (39)
7-9	19.2 (23)
10-12	19.2 (23)

§ “Other” category comprises Bosnia and Herzegovina (2), Cyprus (1), Croatia (1), Greece (2), Israel (3), Italy (4), Kosovo (4), Poland (2), Romania (1), Serbia (2), Spain (2), UAE (7), and Ukraine (7).

The women were asked about their experiences of 15 symptoms in the past 2 weeks and to rate how much these symptoms had bothered them. Only 3.3% of women reported being free of symptoms in the past 2 weeks. Approximately two thirds of women reported suffering from between 1 and 5 symptoms concurrently (67.5%), and a further third reported suffering from 6 or more symptoms concurrently (29.1%). Headaches (61.7%), stomach pain (60.9%), memory problems (44.2%) and back pain (42.5%), loss of appetite (35%), and tooth pain (35%) were amongst the most commonly reported symptoms (Table 1). These were also the symptoms which women were mostly likely to report being “quite a bit”, or “extremely” bothered or caused pain by (Table 2).

**Table 2:** Prevalence and severity of physical health symptoms among trafficked Moldovan women (n = 120)

	Prevalence*	**Symptom Severity n (%)**		
**Symptom**	**% (n)**	**A Little**	**Quite a Bit**	**Very Much**
		**% (n)**	**% (n)**	**% (n)**
**Constitutional**				
Loss of appetite	35.0 (42)	12.5 (15)	15.0 (18)	7.5 (9)
Weight loss	25.8 (31)	6.7 (8)	19.2 (23)	-
Memory problems	44.2 (53)	2.5 (3)	27.5 (33)	14.2 (17)
**Neurological**				
Headaches	61.7 (74)	22.5 (27)	18.3 (22)	20.8 (25)
**Dermatological**				
Skin Problems	9.2 (11)	5.0 (6)	4.2 (5)	-
**Gastrointestinal**				
Stomach Pain	60.9 (73)	15.8 (19)	33.3 (40)	11.7 (14)
**Urogenital**				
Gynaecological	13.3 (16)	8.3 (10)	5.0 (6)	-
Urination Pain	11.7 (14)	3.3 (4)	8.3 (10)	-
**Musculoskeletal**				
Back pain	42.5 (51)	10.0 (12)	20.0 (24)	12.5 (15)
Tooth pain	35.0 (42)	5.8 (7)	27.5 (33)	1.7 (2)
Injuries	6.7 (8)	3.3 (4)	3.3 (4)	-
**Eye/Ear**				
Vision problems	12.5 (15)	10.8 (13)	0.8 (1)	0.8 (1)
Ear pain	5.0 (6)	2.5 (3)	2.5 (3)	-
**Cardiovascular**				
Breathing Difficulties	23.3 (28)	8.3 (10)	13.3 (16)	1.7 (2)
Heart/Chest Pain	24.2 (29)	7.5 (9)	13.3 (16)	3.3 (4)

* Symptoms were coded as present if women reported that they were bothered or caused pain by them “a little”, “quite a bit” or “extremely”.

Variation in the 4 most commonly reported symptoms (headache, stomach pain, memory problems and back pain) was not significantly associated with destination country or the number of months since return to Moldova (Table 3). Women aged 31-44 had higher odds of reporting back pain than women aged 18-20 (p = 0.036), but our analyses detected no other associations between age group and prevalence of physical health symptoms. Duration of exploitation, however, was strongly associated with the prevalence of headache (OR 1.18, 95% CI 1.08-1.30, p<0.001) and memory problems (OR 1.09, 95% CI 1.02-1.17, p = 0.012). Borderline results were returned in respect of stomach pain (OR 1.06, 95% CI 0.99-1.30, p = 0.122) and back pain (OR 1.05, 95% CI 0.98-1.12, p = 0.165). Comparing the prevalence of reported symptoms indicated possible differences between the health profiles of women trafficked for sexual exploitation and women trafficked for labour exploitation, but results were not significant. Sex-trafficked women reported higher prevalence of headache (65.0% vs. 47.8%, p = 0.133) than labour-trafficked women. In contrast, labour-trafficked women reported a higher prevalence of back pain (38.1% vs. 60.9%, p = 0.052) (Table 2). Further analyses showed that sex-trafficked women also reported a higher prevalence of gynecological problems (16.5% vs. 0%) and weight loss (28.9% vs. 13.0%) than labour-trafficked women, whereas labour-trafficked women reported a higher prevalence of vision problems (21.7% vs. 10.3%) (data not shown).

**Table 3:** Distribution of major physical health symptoms among trafficked Moldovan women by selected socio-demographic and trafficking characteristics (n = 120)

	**Headache**		**Memory Problems**		**Stomach Pain**		**Back Pain**	
	**% (n)**	**OR (95% CI)**	**% (n)**	**OR (95% CI)**	**% (n)**	**OR (95% CI)**	**% (n)**	**OR (95% CI)**
**Age group**								
18-20	57.1 (12)	-	52.3 (11)	-	66.7 (14)	-	38.1 (8)	-
21-25	64.4 (38)	1.36 (0.49-3.75)	40.7 (24)	0.62 (0.23-1.70)	57.6 (34)	0.68 (0.24-1.93)	33.9 (20)	0.83 (0.30-2.34)
26-30	71.4 (10)	1.88 (0.44-7.96)	50.0 (7)	0.91 (0.23-3.52)	71.4 (10)	1.25 (0.29-5.45)	35.7 (5)	0.90 (0.22-3.68)
31-44	53.8 (14)	0.86 (0.27-2.79)	42.3 (11)	0.67 (0.21-2.12)	57.7 (15)	0.68 (0.21-2.25)	69.2 (18)	3.65 (1.09-12.29)*
**Destination**								
Turkey	65.2 (30)	-	41.3 (19)	-	56.5 (26)	-	41.3 (19)	-
Russia	64.7 (22)	0.98 (0.39-2.48)	47.0 (16)	1.26 (0.52-3.09)	58.8 (20)	1.10 (0.45-5.70)	38.2 (13)	0.88 (0.36-2.18)
Other§	55.0 (22)	0.65 (0.27-1.56)	45.0 (18)	1.16 (0.49-2.74)	67.5 (27)	1.60 (0.66-3.86)	47.5 (19)	1.29 (0.55-3.02)
**Duration of exploitation (mths)**								
1-3	36.3 (4)	1.18 (1.08 – 1.30) ***	36.3 (4)	1.09 (1.02-1.17)*	45.4 (5)	1.06 (0.99-1.3)	36.3 (4)	1.05 (0.98-1.12)
4-6	46.4 (13)		35.7 (10)		50.0 (14)		25.0 (7)	
7-12	62.5 (30)		33.3 (16)		62.5 (30)		47.9 (23)	
12-24	80.0 (24)		73.3 (22)		73.3 (22)		53.3 (16)	
>24	100.0 (3)		33.3 (1)		66.7 (2)		33.3 (1)	
**Type of exploitation**								
Labour	47.8 (11)	-	39.1 (9)	-	56.5 (13)	-	60.9 (14)	-
Sexual	64.9 (63)	2.02 (0.81–5.06)	45.3 (44)	1.29 (0.52-3.27)	61.9 (60)	1.25 (0.50-3.13)	38.1 (37)	0.40 (0.16-1.01)
**Time since return to Moldova (mths)**								
1-3	62.8 (22)	1.01 (0.90-1.14)	48.6 (17)	0.99 (0.88-1.11)	51.4 (18)	1.10 (0.98-1.24)	31.4 (11)	1.06 (0.94-1.18)
4-6	61.5 (24)		46.2 (18)		59.0 (23)		41.0 (16)	
7-9	52.2 (12)		30.4 (7)		73.9 (17)		65.2 (15)	
10-12	69.6 (16)		47.8 (11)		65.2 (15)		39.1 (9)	

*** p<0.001, ** p<0.01, * p<0.05.

§ “Other” category comprises Bosnia and Herzegovina (2), Cyprus (1), Croatia (1), Greece (2), Israel (3), Italy (4), Kosovo (4), Poland (2), Romania (1), Serbia (2), Spain (2), UAE (7), and Ukraine (7).

## Discussion

Evidence on the health problems experienced by trafficked women who access post-trafficking support services is extremely limited. Although not all trafficked women will choose to register with post-trafficking programmes, or indeed be offered the opportunity to do so, data on the needs of those trafficked women who do access support are urgently needed in order to inform the provision of policies and services. Our results demonstrate a substantial burden of poor physical health amongst trafficked women who registered with the IOM for post-trafficking support services in Moldova. Headache (61.7%), stomach pain (60.9%), memory problems (44.2%) and back pain (42.5%) were particularly prevalent. Between a quarter and a third of women also reported suffering loss of appetite (35.0%), tooth pain (35.0%), weight loss (25.8%), chest pain (24.2%), and breathing difficulties (23.3%). The high prevalence of headache, memory loss, poor appetite and pain in our sample may be explained at least partly by the women’s poor mental health. As described elsewhere, 88% of women in this sample met ICD-10 criteria for mental disorder [19,24]. We note, however, that these symptoms may be due to other pathologies and that chronic pain (arising from such untreated or unrecognized pathologies) can itself contribute to poor mental health. Poor health outcomes were not limited to women trafficked for sexual exploitation but were also reported by women trafficked for labour exploitation.

Our findings support those of Zimmerman et al, whose multi-country study also reported that trafficked women being assisted by post-trafficking support services suffered from high levels of headache, memory problems, and stomach and back pain [8]. However, whereas Zimmerman et al reported on women interviewed between 0 and 14 days after entry into post-trafficking support services, the trafficked women in our sample had received, on average, 6 months of post-trafficking support. Despite this longer period of support, women continued to report high levels of headache, memory problems, and stomach and back pain. Post-trafficking programs working with women who have returned to their country of origin may encompass initial “crisis-intervention” work (i.e., meeting urgent needs, ensuring safety) and, later, “reintegration” support (i.e., longer-term recovery) [12]. Our findings suggest that many of the trafficked women who register for such support have symptoms that may be indicative of physical health problems and psychological distress and that should be attended to not only during crisis support but also throughout reintegration-stage programming. Furthermore, the range of symptoms reported suggests that if the needs of these women are to be met, more comprehensive provision of medical care, psychological support and counselling is required.

Several studies have reported a high prevalence of physical and sexual violence among trafficked women. Although there has been limited research on the long-term physical health consequences of violence and abuse, a number of studies conducted with women who have experienced domestic violence have shown the prevalence of a number of physical health symptoms (including headache, back pain, abdominal pain, loss of appetite, and gynaecological and urinary problems) to be significantly higher among female victims of domestic abuse than among non-abused women [25-29]. The prevalence of physical symptoms among our sample of trafficked women was comparable to or higher than has been reported for other groups of abused women [25,27,28]. A study using the Miller Abuse Physical Symptom and Injury Scale with women who had experienced intimate partner violence, for example, found that headache was reported by 48% of women, back pain by 40%, stomach pain by 22% and loss of appetite by 9% [25]. The conditions in which trafficked women live and work may likely to contribute to poor physical health. Living and working conditions that are overcrowded, poorly ventilated and lacking adequate sanitation may increase the risk of infectious and communicative disease; long working hours, little rest time and inadequate nutrition may result in fatigue, weight loss and increased vulnerability to infection; and receiving inadequate safety training and working without protective equipment and is likely to increase occupational risks.

Our study findings must be interpreted within the context of a number of limitations. Ambiguities in the UN definition of human trafficking – regarding, for example, the meanings of exploitation, consent and coercion – mean that identifying women who have been trafficked is challenging [30,31] and that the distinctions drawn between trafficking and other forms of migrant exploitation and between trafficking and migrant sex work are often contentious [32-35]. For researchers, these conceptual difficulties can translate into problems in deciding who should be included within studies on human trafficking [36,37]. In this study, assessments of whether or not women had been trafficked were made by IOM case managers. These assessments were made independently of the research and preceded the onset of the study. When assessing whether or not a person had been trafficked, IOM case managers used standardised screening questionnaires that included questions about women’s trafficking experiences, including their recruitment, transportation and exploitation. Alternative programmes of IOM assistance were available to other groups of vulnerable migrants who had returned voluntarily to Moldova but who had not been trafficked. IOM also provides assistance to people who have been deported following an illegal stay overseas.

The extent to which our findings are generalisable beyond trafficked women within post-trafficking support settings is unclear. We were able to recruit 68% of consecutive women returning to Moldova through the IOM assistance programme and our analysis of IOM data suggested that our sample was reasonably representative of the women with whom they work. We were, however, unable to ascertain whether the trafficked women who registered for support with the IOM assistance programme differed from the trafficked women who did not, including in relation to health status. We are not aware of evidence of if or how trafficked people’s engagement with post-trafficking support services varies as a function of their health. Research has shown, however, that trafficked women with more dysfunctional family relationships and poorer social support networks (both factors which could contribute to psychological distress) are more likely to engage with support services [38]. Similarly, research with domestically abused women has found that most victims first seek help from family and friends rather than turning to shelters and advocacy organisations [28,39,40]. If women with a higher level of need were more likely to be in contact with the IOM our study may overestimate the prevalence of health symptoms compared to the wider population of trafficked persons. Conversely, if the women included in the study represented individuals who were less distressed and more able to contact and use services, our study may underestimate symptom prevalence.

A further limitation of the study is that we were unable to control for women’s pre-trafficking physical and mental health status and are therefore unable to determine whether women’s health problems developed as a direct result of trafficking. Women’s physical health symptoms may have existed prior to, or have been exacerbated by, their experiences whilst trafficked. Indeed the women in our sample reported a high prevalence of pre-trafficking problems, such as low socio-economic status and child abuse, which may have increased their vulnerability to poor physical health [41,42]. Information was also not available on the length or type of support that women received prior to returning to Moldova; emergency medical care and counselling are available in countries with IOM missions or partner organisations, but it remains the case that many trafficked people have limited access to services prior to repatriation. Nonetheless, our results emphasise the importance of post-trafficking support services to attend to the health needs of their service users.

Finally, our study, which was designed to describe the prevalence of health problems among trafficked women receiving post-trafficking support in Moldova, was underpowered to detect differences in health risks between socio-demographic groups or in relation to the characteristics of women’s trafficking experiences. Our analysis did, however, identify that duration of exploitation was strongly associated with the prevalence of headache and memory problems in our sample. Women who are trafficked for longer periods may be exposed to a greater number of health risks and episodes of abuse and, subsequently, may experience poorer physical health. The higher prevalence of headache and memory problems may also be related to poorer mental health amongst women who have been exploited for longer periods. Hossain et al have previously demonstrated, for example, that symptoms of depression and anxiety are associated with duration of exploitation amongst trafficked women receiving post-trafficking support [15]. These findings suggest that trafficked women who access post-trafficking support services after longer periods of exploitation may require longer-term or more intensive medical care and psychological support.

To our knowledge this was the first study to collect quantitative data on the physical health symptoms reported by women accessing post-trafficking support services following trafficking for labour exploitation. The harms caused by trafficking for labour exploitation have received much less attention to date than those caused by sexual exploitation, and the health needs of people trafficked for labour exploitation have been neglected as a topic of research. Our results demonstrate, however, a high prevalence of physical symptoms among 23 women who were receiving post-trafficking support after being exploited in domestic work, agricultural labour and begging. Furthermore, the wide differences between women who had been trafficked for sexual exploitation and women who had been trafficked for labour exploitation in the prevalence of symptoms such as weight loss, gynaecological problems, back pain, and vision problems points to the likelihood that a larger study would have detected significant differences in the physical health profiles of these women. Such differences could have important implications for the planning and provision of support and assistance to women trafficked for labour exploitation. Although the IOM estimates that approximately 33% of victims of trafficking are trafficked for labour exploitation [5], expertise in caring for trafficked people has been developed primarily in relation to trafficking for sexual exploitation. In order to assess the extent to which existing guidance and best practice is relevant for women trafficked for labour exploitation, further research with larger samples is needed to investigate the health risks and outcomes associated with trafficking for labour exploitation.

The difficulties inherent in identifying cases of human trafficking mean that accurate data on the scale of the problem are difficult to obtain [43]. As discussed above, not only researchers but also service providers and officials face considerable challenges in interpreting and applying the UN definition of human trafficking. Even when trafficked people are no longer in a situation of exploitation, their abuse may go undocumented if they are unaware of how to report it or choose not to do so, for instance because of fear of harm towards themselves or their families or because they do not trust the ability or willingness of the authorities to assist them [44-46]. Nonetheless, our results demonstrate a substantial burden of poor physical health among trafficked women who were known to and had registered with the IOM Moldova Assistance and Protection Programme in 2008 and highlight the importance of providing medical, psychological and dental care within post-trafficking support programs.

## Conclusions

Policymakers and practitioners should be aware that, after returning to their countries of origin, many of the trafficked women who register with post-trafficking support programs may be suffering from a variety of physical and psychological health problems. Services for trafficked women who have returned to their countries of origin should, in both the “crisis” and “reintegration” phases of their support programs, include comprehensive medical, psychological and dental care. Health problems are not limited to women who access support after having been trafficked for sexual exploitation but are also experienced by those women who register for assistance after being exploited in a variety of labour settings. It is vital that anti-trafficking policies and programs now expand their focus beyond trafficking for sexual exploitation and recognize and provide for the needs and vulnerabilities of victims of labour exploitation.

## Competing interests

The authors declare that they have no competing interests.

## Authors’ contributions

NO and MA were responsible for designing the study and selecting study instruments with support from MH. NO, LG, VG, and CT collected the data. MA and MH supervised the study. SO and MA analyzed the data. SO conceived and wrote the first draft of the paper. All authors approved the final draft.

## Pre-publication history

The pre-publication history for this paper can be accessed here:

http://www.biomedcentral.com/1472-6874/12/20/prepub

## Supplementary Material

Additional file 1 **Physical Health Questionnaire.** Description of data: Questionnaire used to measure the self-reported prevalence and severity of physical health symptoms. Click here for file
