# Supplementary material for: Physical health symptoms reported by trafficked women receiving post-trafficking support in Moldova: prevalence, severity and associated factors
Source: BMC Womens Health. 2012 Jul 26;12:20. doi: 10.1186/1472-6874-12-20 (PMC3460745; doi:10.1186/1472-6874-12-20)
Supplement: Additional file 1 — Physical Health Questionnaire. Description of data: Questionnaire used to measure the self-reported prevalence and severity of physical health symptoms. [file 1472-6874-12-20-S1.pdf]

Physical Health Questionnaire

The physical health question was derived from the Miller Abuse Physical Symptoms and Injury Survey [22]. Women were asked whether, in the last two weeks, they had experienced any of the following symptoms:

1. Headaches
2. Loss of appetite
3. Significant weight loss
4. Difficulty remembering things
5. Difficulty with your eyes or seeing
6. Difficulty with your ears or with hearing, ear pain or injury
7. Difficulty with your teeth or mouth
8. Any injuries (e.g., lack eyes, bruises, bloody nose, jaw injury)
9. Breathing problems
10. Chest pain, heart pain, or heart palpitations
11. Pain in your stomach/abdomen
12. Painful urination
13. Gynaecological infections or symptoms of infection
14. Back pain
15. Skin problems

For each question, if women answered yes, they were asked to indicate to what degree the symptom had bothered them or caused them pain (not at all, a little, quite a bit, or very much).
